# Effects of Stress in Adaptation to Undergraduate Life on Psychiatric Morbidity: Mediating Effects of Early Trauma and Adverse Family Factors

**DOI:** 10.3389/fpsyt.2022.538200

**Published:** 2022-04-08

**Authors:** Yingzhe Zhang, Jeremy Coid, Xiang Liu, Yamin Zhang, Huan Sun, Xiaojing Li, Wanjie Tang, Qiang Wang, Wei Deng, Liansheng Zhao, Xiaohong Ma, Yajing Meng, Mingli Li, Huiyao Wang, Ting Chen, Qiuyue Lv, Wanjun Guo, Tao Li

**Affiliations:** ^1^Mental Health Center and Psychiatric Laboratory, The State Key Laboratory of Biotherapy, West China Hospital of Sichuan University, Chengdu, China; ^2^West China Brain Research Center, West China Hospital of Sichuan University, Chengdu, China; ^3^Mental Health Education Center, Sichuan University, Chengdu, China; ^4^West China School of Public Health and West China Fourth Hospital, Sichuan University, Chengdu, China; ^5^Centre for Psychology Education and Consultation, Sichuan University, Chengdu, China; ^6^Hangzhou Seventh People's Hospital and Affiliated Mental Health Center, Zhejiang University School of Medicine, Hangzhou, China

**Keywords:** undergraduate students, common mental health disorders, adaptation, stress, childhood maltreatment

## Abstract

**Purpose:**

University students experience high levels of stress, and the prevalence of depression is higher than in the general population. The reason is not clear. More effective interventions and better prevention are needed.

**Methods:**

We did annual cross-sectional surveys of Chinese undergraduates 2014–2018 (mean age 18.7 [SD 2.1], *N* = 39,573). We measured adaptation to university life using the Adolescent Self-rating Life Events Checklist (ASLEC) and common mental disorders using standardized self-report instruments. Regression analyses identified associations between childhood maltreatment, current family problems, stress in adaptation to undergraduate life, and psychiatric morbidity. Mediation analyses further tested relationships between these factors.

**Results:**

Childhood maltreatment, current family problems, stress in adaptation, and psychiatric morbidity were all significantly associated with each other. The strongest association between childhood experiences and psychiatric morbidity was for sexual abuse and depression (OR = 3.39, 95%CI: 2.38–4.83, *p* < 0.001) and between stress from adaptation and somatic disorder (OR = 4.54, 95%CI: 3.62–5.68, *p* < 0.001). Associations between childhood maltreatment and stress from university life were partly mediated by psychiatric morbidity. Associations between family problems and psychiatric morbidity were mediated by stress from university life.

**Conclusions:**

Stress from adaptation to university life and pressures from academic study exert stronger effects on psychiatric morbidity among students than childhood traumatic experiences and current family problems, although these factors are closely interrelated. Mental health services for students should focus on adaptation to university life and pressures from academic study as well as external factors of childhood trauma and family problems.

## Introduction

Research into the mental health of undergraduates has shown that psychiatric morbidity, including depression and suicidal ideation, is higher than in the general population (1–3). Meta-analysis of studies of students in different countries found a weighted prevalence of 30.6% with depression (2) and among US medical students a prevalence of 27.2%, with 11.1% of the latter reporting suicidal ideation (3). A meta-analysis of studies of Chinese university students found a similar overall prevalence of 28.4% with depression, and with an earlier meta-analysis of suicidal ideation showing a pooled prevalence of 10.7% (4). There have been concerns that psychiatric morbidity among students may be increasing (5). College students are at a period of transition from childhood to adulthood during which most are no longer under parental supervision. They experience major life changes, intense competition to succeed, and economic pressures due to increased expenses and tuition fees. They also enter a time period when certain mental disorders are most likely to show first presentation (6, 7).

Although students in China tend to come from wealthier families, they can still experience childhood disadvantage and maltreatment. Maltreatment in childhood is recognized as an important risk factor for mental health problems worldwide (8), including anxiety, depression, and suicidal behavior (9–11). Another form of childhood disadvantage common in China is being left behind by parents during childhood. China has undergone the most rapid process of urbanization since the mid-twentieth century and migrant workers have driven China's economic growth, constituting more than one-third of the working population (12). Nearly 70% of the children of migrant workers do not move with their parents due to financial constraints and the nature of transient work in urban areas (13). The majority of these children are left behind in the countryside and raised by grandparents or move between caregivers, with varying levels of contact with parents. Meta-analysis has shown that left behind children have higher rates of depression, anxiety, suicidal ideation, conduct disorder, substance use, wasting, and stunting (14). Studies show that negative life events involving disadvantage and maltreatment during childhood increase the risk of mental health problems in adulthood, as well as increasing likelihood of encountering stressful events later in life (15, 16).

At the same time, negative life events which have occurred recently during early adulthood can also be strong risk factors for psychopathology (17, 18) and their number and severity can predict severity and the likelihood of repetition of suicidal behavior (19). However, few studies have investigated the combined effects of these two key areas of exposure (childhood disadvantage/maltreatment and stressful negative life events due to family problems in early adulthood) on psychiatric morbidity among students. According to the stress-diathesis model, their psychopathology should be influenced not only by the independent effects of the diathesis of childhood adversity but also by interactions with more recent stressful life events, resulting in multiplicative effects (20, 21), and for students this can include stress from difficulty in adaptation to university life and studies.

Students who fail to adapt to the challenges of college life may have difficulties with educational performance and even drop out of college. They may react with severe stress to these challenges and have interpersonal difficulties with fellow students and teachers. The effects of stress may lead to symptoms of psychopathology, including anxiety and depression. On the other hand, the relationship with psychopathology and stress may be bi-directional, and some students may be unable to adapt because of pre-existing psychopathology. Most colleges provide specialist health facilities for students, although these vary in healthcare coverage. Because a primary aim of health services to students is to maintain optimum functioning in their studies, it is important to differentiate between the stressful effects of poor adjustment to college life, which are largely due to study pressures, and those which result from pre-existing problems. The latter can include early childhood trauma and disadvantage together with ongoing stress from family problems. All these factors can result in undergraduates being more vulnerable to stress from their studies, having difficulties in adapting to college life, and increasing their risk of psychopathology. By definition, in the first year of attendance at university, these factors of experiences in childhood and current negative family experiences are problems that the students bring with them to university life and which may impact their adaptation to university and success in their studies. Unless symptoms are sufficiently severe to constitute mental disorders, the first line of intervention is likely to be counseling or psychotherapy for a young person rather than a formal referral to mental health services. It is therefore essential to target the key issues that require resolution in any psychotherapeutic intervention but also to be clear about the main presenting problems. The strategy for therapeutic intervention may be to focus on addressing issues students bring with them to university, including childhood maltreatment and distress from unresolved family difficulties beyond their control.

On the other hand, it is possible that difficulty in adaptation to the first year of university life occurs because they find their courses highly challenging. University entry is highly competitive in China, and Chinese students outperform those in many countries on standardized exams. It has been argued that this high level of achievement comes at a price, and Chinese students report some of the highest stress levels in the world, which can in turn lead to debilitating mental health outcomes, with feelings of despair, adversarial feelings of competitiveness with peers, and heightened anxiety (22). Female students experience more stress and the combination of heightened emphasis on academic achievement in rural areas but fewer resources than urban schools are associated with higher stress levels. Feeling disconnected from college due to lack of close friends or poor relationships with teachers is associated with higher student stress levels, and conflicts with peers is closely linked with higher levels of academic stress (22). This would suggest that specialist student healthcare professionals may need to become aware and alert policymakers and university authorities of these risks, with a specific need to be aware of the college environment, including inherent study pressures related to their courses.

Relationships between child maltreatment and later life difficulties are complex and can involve multiple pathways. The relationship between child maltreatment and substance misuse in adulthood has previously been investigated using interaction analysis (23). Among university students, the association with poor mental health outcomes have been investigated using mediation analysis (24). We considered that factors such as child maltreatment in our sample and ongoing family problems at the time of university entry could lead to stress and failure to adapt to university life and studies, but also to psychopathology among students. These external factors involving the family at different life stages could lead directly to adaptation stress and psychopathology but also could involve interactions between the two, leading to multiplicative effects on stress and psychopathology as outcomes. However, there are other potential pathways involving direct effects of adaptation stress on psychopathology (or vice versa) which could be more powerful, particularly when interacting with early or ongoing family problems. In addition, although cross-sectional studies cannot demonstrate causation, the possibility remains that complex causal pathways exist and that the relationships between childhood maltreatment and adverse outcomes at university are mediated by factors such as ongoing family problems. Furthermore, the pathway to poor adaptation could be mediated by psychopathology and vice versa.

The overarching aim of this study was to inform student health services on interventions for stress and poor adaptation among students in their first year of university life. The specific aims of the study were firstly, to explore associations between demographic factors and stress from life events to identify characteristics of students at greatest risk. We secondly aimed to investigate whether interventions should focus primarily on early experiences of childhood maltreatment and disadvantage or whether stress was primarily due to current family problems. We considered these two sources of potential poor adaptation to be external factors to university life and problems that the students had brought with them to university. We thirdly investigated the effects of psychiatric morbidity at the time of the survey on self-reported stress and poor adaptation to university life. However, we also investigated bi-directionality and whether poor adaptation to university life and high levels of stress had increased risk of psychiatric morbidity in the students. Finally, we aimed to investigate interaction effects and potential mediation pathways between child maltreatment and disadvantage and current family problems and both stress at university due to poor adaptation, and psychiatric morbidity. We hypothesized that there would be interaction effects of childhood maltreatment and family problems on stress at university. Also, we believed that early experiences of maltreatment and disadvantage and stress at university would have interaction effects on psychiatric morbidity. We also hypothesized that stress at university would partly mediate the association between early childhood disadvantage and psychiatric morbidity.

## Methods

### Participants

The University Students Study of Sichuan Province is an ongoing investigation into mental health problems associated with student life, risk factors preceding university entry, and their impact on academic performance and mental health at a comprehensive university in southwest China. All participants were invited to take part in the annual survey in 2014–2018 and complete a questionnaire online 1–3 months after university entry. This cross-sectional study sample between 2014 and 2018 was used in this analysis which included an overall total of 46,774 potential voluntary respondents. Following a quality control step excluding those who gave incomplete information, 39,573 were included, an 84.6% response rate.

The study was approved by the Medical Ethics Committee of West China Hospital of Sichuan University.

### Survey Measurements

#### Stressful Life Events

We measured adaptation according to (i) the level of stress reported by students in their first months of study in a Chinese comprehensive university and (ii) a count of the number of incidents reported. The Adolescent Self-rating Life Events Checklist (ASLEC) was used to assess the intensity and number of psychological stressors experienced by students (25). In the present study, 16 major negative life events relevant to current undergraduate life were selected from the original 27 life events considered by ASLEC. These included items of difficulties with their studies, interpersonal problems with other students and staff, and other problems of adaptation including difficulty being separated from family, disliking college and a new lifestyle, and wanting to change their course or leave college. In this study, we considered high scores on these items and total number of items as two separate measures of poor adaptation to and difficulty coping with university life in our analyses. (For scores, the respondent rated the psychological impact of the life event (0 = *not occur*; 1 = *no effect*; 2 = *mild*; 3 = *moderate*; 4 = *severe*; 5 = *extremely severe*) (26). An overall college life-event related stress score was the simple sum of the 16 separate stress scores (range = 0–80).

#### Psychiatric Morbidity

We used standardized cut-off scores to create binary variables of three conditions: depression, somatic disorder, and obsessive-compulsive disorder. The PHQ-9 Depression module of the Prime-MD diagnostic instrument for common mental disorders (27) measured a probable depressive disorder over the past 2 weeks with a range from 0 to 27. In our study, we set the cut-off at 15 and above based on a previous meta-analysis, indicating severe depression (28).

The PHQ-15 module of somatic symptoms provided a continuous score ranging from 0 to 30 over the past month, with a cut-off at 10 rating a binary diagnosis of moderate or severe somatization disorder (29). The Obsessive-Compulsive Inventory-Revised (OCI-R) (30) rated current obsessional and compulsive symptoms and provided a continuous measure, ranged from 0 to 72, with cut-off of 21 for probable obsessive-compulsive disorder. Participants additionally reported whether they had ever attempted suicide.

#### Childhood Maltreatment

Participants were asked if they had ever been physically abused, neglected, or sexually abused by rating as *never, occasionally, sometimes, often*, and *always*, using the Childhood Section of the Chinese World Mental Health Initiative Composite International Diagnostic Interview (31, 32). Specifically, being physically abused before age 16 sometimes or more was labeled as physical abuse, being sexually abused occasionally or more often was labeled as sexually abused, being taken care of and protected occasionally or less was labeled as neglect. Additionally, participants were asked how long they were separated from their parents constantly before age 16. Six months or more was labeled as left behind children. In our analysis, we have a potential count variable for childhood maltreatment based on these categorical variables from 0 to 4.

#### Current Family Problems

We separated four items (serious illness in family, death of relative, family financial problems, conflict between parents) from the other ASLEC items because they were considered potential determinants of family problems. We did not use scores of stress due to these items which were binary in our analyses or a count of the items with a maximum of 4. They were therefore no longer included as outcome but were used as predictor variables in our analyses.

### Analysis

We initially investigated associations between demography and ratings on stressful events associated with adaptation problems to first year university life. We measured the associations between the occurrence of any stressful life event using odds ratios, and the impact of the life event on measures of stress using coefficients. We next tested independent associations between childhood maltreatment and current family problems, with ratings of stressful university events as outcome, using linear regression. We tested independent associations between stressful problems/events at university measured using counts of the number of events and psychiatric morbidity measured using binary variables for presence or absence of each condition, in logistic regression.

To better understand mechanisms of childhood maltreatment/disadvantage and family problems on psychiatric morbidity and stress from university life, we carried out interaction analyses between these factors, exploring effect modification. We applied linear regression with counts of childhood maltreatment/disadvantage experiences, counts of family problems, and counts of psychiatric morbidities (maximum 4) as independent variables with continuous stress scores as outcome. Similarly, we applied logistic regression with counts of childhood maltreatment/disadvantage experiences (maximum 4), counts of family problems (maximum 4), and continuous stress at university scores as independent variables with psychiatric morbidity (binary variable, i.e., at least one of listed psychiatric morbidity) as outcome.

Psychiatric morbidity was considered as a potential mediator when it demonstrated a significant relationship between both (1) stressful life events related to adaptation at university and (2) childhood maltreatment/disadvantage. We previously found stressful life events related to adaptation measured by the total number of stressful events reported by students significantly relevant between both psychiatric morbidity and childhood maltreatment/disadvantage. In our final analyses, we therefore investigated whether the pathways from childhood maltreatment/disadvantage and family problems to stress resulting in poor adaptation were mediated by psychiatric morbidity and whether the pathway to psychiatric morbidity was mediated by stress from poor adaptation. We followed the bootstrapping mediation analysis protocol of Preacher and Hayes (33). In the structural equation model for mediation analysis, we used latent variables not directly observed but inferred from other variables directly measured through a mathematical model to describe childhood maltreatment/disadvantage, family problems, and psychiatric morbidity (34).

All independent association analyses were performed in SPSS 24. Mediation analyses were conducted in AMOS 24.

## Results

Mean age of the student population was 18.71 years (SD 2.14), range 14–34 years, with 13.7% starting university before age 18 years; 50.5% were male, most Han Chinese (90.0%), with most students' families having high or medium level earnings.

Table 1 shows associations between demography and experiencing problems with adaptation in the first year at university. Younger students were significantly more likely to report occurrence of most individual problems. Those who did rated them significantly more negatively. When compared to female students, men were significantly more likely to report problems with teacher relationships, being criticized/penalized, relationship break-up, fights, and wanting to change major/leave university, but were less likely to report exam failure, heavy study load, argument with classmates, change in living habits, and disliking university. However, they reported being significantly less affected by these experiences than women, except for break-up of relationships. There were no differences between ethnic minorities and Han Chinese majority, except that more minorities found change in living habits more stressful. Students from poorer family backgrounds were less likely to report exam failure and loss of face. They reported less stress from exam failure and heavy study load but greater stress from being ignored or discriminated against by classmates. Table 1 also summarizes these results using scores of numbers of actual events and their associated stress scores. These confirmed that younger age was associated with total study problems/events, interpersonal problems, and adaptation problems. Male students reported fewer study problems and adaptation problems with lower associated stress levels, but more interpersonal problems and higher associated stress scores than female students.

**Table 1:** Univariate associations between demography and negative ratings associated with adaptation problems to first year undergraduate life (*N* = 39,573).

**Study problems**	**Age**		**Sex, male**		**Ethnic minority**		**Low family income**	
	**18.71** **±** **2.14**		***N*** **=** **19,983 (50.5%)**		***N*** **=** **3,954 (10.0%)**		***N*** **=** **6,365 (16.1%)**	
	**OR (95%CI)**	**β (95%CI)**	**OR (95%CI)**	**β (95%CI)**	**OR (95%CI)**	**β(95%CI)**	**OR (95%CI)**	**β (95%CI)**
Failed exam/poor results	0.80^***^ (0.79–0.81)	−0.06^***^ (−0.06–−0.05)	0.84^***^ (0.81–0.87)	−0.08^***^ (−0.10–−0.05)	1.05 (0.98–1.12)	0.05^*^ (0.01–0.09)	0.93^*^ (0.88–0.98)	−0.04^*^ (−0.07–−0.01)
Heavy study load	0.93^***^ (0.92–0.94)	−0.04^***^ (−0.05–−0.03)	0.75^***^ (0.72–0.78)	−0.04^***^ (−0.07–−0.02)	1.06 (1.00–1.13)	0.04^*^ (0.00–0.08)	0.99 (0.93–1.04)	−0.45^***^ (−0.08–−0.01)
Teacher relationship	0.93^***^ (0.92–0.95)	−0.02^***^ (−0.03–−0.01)	1.07^*^ (1.01–1.14)	−0.07^**^ (−0.12–−0.02)	1.01 (0.91–1.12)	0.05 (−0.03–0.13)	0.97 (0.88–1.05)	−0.03 (−0.09–0.04)
Failure achieve award/status	0.93^***^ (0.916–0.943)	−0.04^***^ (−0.05–−0.03)	0.98 (0.94–1.04)	−0.07^***^ (−0.11–−0.04)	1.01 (0.92–1.09)	0.02 (−0.04–0.08)	0.93^*^ (0.87–1.00)	−0.04 (−0.09–0.01)
Criticized/penalized	0.92^***^ (0.908–0.943)	−0.04^***^ (−0.05–−0.02)	1.23^***^ (1.15–1.31)	−0.16^***^ (−0.21–−0.10)	1.03 (0.93–1.15)	−0.05 (−0.14–0.04)	0.99 (0.90–1.08)	−0.02 (−0.10–0.05)
**Interpersonal problems**								
Classmate ignore/discriminate	0.94^***^ (0.93–0.95)	−0.05 (−0.06–−0.04)	1.01 (0.96–1.05)	−0.10^***^ (−0.13–−0.07)	0.97 (0.90–1.05)	0.00 (−0.05–0.06)	1.01 (0.95–1.08)	1.66^***^ (1.65–1.68)
Argument with classmates/friend	0.88^***^ (0.87–0.89)	−0.06^***^ (−0.07–−0.05)	0.81^***^ (0.78–0.85)	−0.11^***^ (−0.14–−0.08)	1.03 (0.96–1.11)	−0.01 (−0.06–0.04)	0.96 (0.90–1.01)	−0.02 (−0.06–0.02)
Relationship break-up	0.99 (0.98–1.01)	−0.07^***^ (−0.08–0.06)	1.64^***^ (1.55–1.73)	0.18^***^ (0.13–0.23)	1.07 (0.98–1.17)	−0.04 (−0.12–0.04)	0.95 (0.89–1.02)	0.02 (−0.05–0.09)
Public loss of face	0.87^***^ (0.86–0.88)	−0.05^***^ (−0.06–−0.04)	1.01 (0.97–1.06)	0.05^**^ (0.02–0.08)	1.04 (0.96–1.12)	0.01 (−0.04–0.06)	0.90^**^ (0.85–0.96)	−0.01 (−0.05–0.03)
Physical fight(s)	0.99 (0.96–1.02)	0.02 (−0.00–0.04)	2.27^***^ (1.99–2.57)	−0.18^**^ (−0.30–−0.05)	1.09 (0.90–1.32)	−0.10 (−0.28–0.08)	1.08 (0.93–1.27)	0.03 (−0.11–0.18)
Change in living habits	0.88^***^ (0.87–0.89)	−0.04^***^ (−0.05–−0.03)	0.83^***^ (0.80–0.86)	−0.04^***^ (−0.07–−0.02)	1.07^*^ (1.00–1.14)	0.02 (−0.02–0.06)	0.96 (0.91–1.01)	−0.02 (−0.05–0.01)
Dislike university	0.93^***^ (0.92–0.94)	−0.03^***^ (−0.04–−0.02)	0.77^***^ (0.73–0.81)	−0.07^***^ (−0.10–−0.03)	1.076 (0.996–1.162)	0.026 (−0.030–0.081)	0.99 (0.93–1.06)	−0.03 (−0.07–0.02)
Family separation stress	0.89^***^ (0.88–0.90)	−0.04^***^ (−0.04–−0.03)	0.71^***^ (0.69–0.74)	−0.11^***^ (−0.13–−0.09)	1.01 (0.94–1.08)	0.03 (−0.01–0.06)	1.05 (0.99–1.10)	−0.01 (−0.04–0.02)
Change major	0.97^**^ (0.94–0.99)	0.00 (−0.02–0.02)	1.17^***^ (1.08–1.28)	−0.02 (−0.11–0.07)	1.10 (0.96–1.26)	−0.00 (−0.15–0.14)	1.04 (0.93–1.17)	0.01 (−0.11–0.123)
	β(95%CI)	β(95%CI)	β(95%CI)	β(95%CI)	β(95%CI)	β(95%CI)	β(95%CI)	β(95%CI)
Total study problems/events	−0.08^***^ (−0.09–−0.07)	−0.16^***^ (−0.18–−0.14)	−0.09^***^ (−0.12–−0.06)	−0.09^**^ (−0.16–−0.02)	0.03 (−0.00–0.07)	0.12^*^ (0.01–0.23)	−0.04 (−0.07–0.00)	−0.16^***^ (−0.26–−0.07)
Total interpersonal problems/events	−0.05^***^ (−0.06–−0.05)	−0.12^***^ (−0.14–−0.01)	0.05^***^ (0.03–0.07)	0.21^***^ (0.13–0.28)	0.02 (−0.02–0.06)	0.01 (−0.13–0.13)	−0.03 (−0.06–0.00)	−0.05 (−0.16–0.06)
Total adaptation problems/events	−0.06^***^ (−0.07–−0.06)	−0.10^***^ (−0.11–−0.08)	−0.17^***^ (−0.19–−0.14)	−0.18^***^ (−0.23–−0.13)	0.04^*^ (0.00–0.08)	0.06 (−0.02–0.15)	0.00 (−0.03–0.03)	−0.04 (−0.11–0.03)
All problems/events	−0.20^***^ (−0.21–−0.18)	−0.36^***^ (−0.40–−0.32)	−0.21^***^ (−0.27–−0.14)	−0.19^**^ (−0.33–−0.05)	0.09 (−0.01–0.20)	0.19 (−0.04–0.43)	−0.06 (−0.15–0.02)	−0.22^*^ (−0.42–−0.03)

* *p < 0.05,*

** *p < 0.01,*

*** *p < 0.001*.

Table 2 shows independent associations between childhood disadvantage and summary stress scores for problems/events ratings during the first year of undergraduate life. All experiences of childhood trauma were significantly associated with stress scores for study, interpersonal, adaptation problems, and their total scores. The strongest associations between these childhood experiences were shown for sexual abuse, followed by emotional neglect, physical abuse, and being a left behind child, respectively. Table 2 also shows the independent associations between recent and ongoing family problems on summary stress scores. All experiences/events were significantly associated with stress scores in domains of study, interpersonal, adaptation problems, and total scores. Conflict between parents was most strongly associated with stress scores in each domain. The other three experiences showed similar impact.

**Table 2:** Independent associations between childhood maltreatment/disadvantage or family problems and stress scores on adaptation during first year of undergraduate life (*N* = 39,573).

	**Study problem events**	**Interpersonal problem events**	**Adaptation problem events**	**All problem Events**
	**β(95%CI)**	**β(95%CI)**	**β(95%CI)**	**β(95%CI)**
**Childhood maltreatment/disadvantages**				
Left-behind child^a^ *N* = 9,471 (23.9%)	0.35^***^ (0.27–0.43)	0.17^***^ (0.08–0.25)	0.06^*^ (0.01–0.12)	0.75^***^ (0.58–0.91)
Sexual abuse^a^ *N* = 1,147 (2.9%)	0.81^***^ (0.62–1.01)	0.89^***^ (0.68–1.11)	0.80^***^ (0.66–0.95)	2.37^***^ (1.97–2.77)
Physical abuse^a^ *N* = 10,339 (26.1%)	0.36^***^ (0.28–0.45)	0.39^***^ (0.29–0.48)	0.32^***^ (0.26–0.39)	0.96^***^ (0.79–1.14)
Emotional neglect^a^ *N* = 13,385 (33.8%)	0.58^***^ (0.50–0.66)	0.47^***^ (0.38–0.56)	0.38^***^ (0.32–0.44)	1.47^***^ (1.31–1.63)
**Family problems**				
Serious illness^b^ *N* = 4,963 (12.5%)	0.77^***^ (0.67–0.87)	0.78^***^ (0.67–0.89)	0.48^***^ (0.40–0.56)	2.07^***^ (1.87–2.27)
Relatives death^b^ *N* = 4,519 (11.4%)	0.78^***^ (0.68–0.88)	0.77^***^ (0.66–0.89)	0.37^***^ (0.29–0.45)	2.02^***^ (1.82–2.23)
Family financial problems^b^ *N* = 11,553 (29.2%)	0.77^***^ (0.70–0.84)	0.60^***^ (0.52–0.68)	0.57^***^ (0.51–0.62)	2.10^***^ (1.95–2.24)
Conflicts between parents^b^ *N* = 10,023 (25.3%)	1.62^***^ (1.55–1.69)	1.22^***^ (1.14–1.31)	0.64^***^ (0.58–0.69)	3.91^***^ (3.76–4.06)

a *Adjusted for sex, age, ethnic minority, low family income, and other disadvantage/maltreatment (binary variables)*.

b *Adjusted for sex, age, ethnic minority, low family income, and Family Problems (binary variables)*.

* p < 0.05, ** p < 0.01,

*** *p < 0.001*.

Table 3 shows the independent associations between childhood maltreatment/disadvantage, recent and ongoing family problems, and psychiatric morbidity, including depression, somatic disorder, obsessive-compulsive disorder, and suicide attempts. All childhood and recent factors were significantly associated with the four categories of psychiatric morbidity except for an association between a relative/friend's death and depression. The strength of association between childhood events and recent/ongoing events differed. Childhood events showed strongest associations with depression followed by suicide attempts. Sexual abuse and emotional neglect in childhood showed strongest associations. Recent events showed strongest associations with somatic disorder and with suicide attempts and obsessive-compulsive disorder showing somewhat stronger associations than depression. Except for depression, conflict between parents showed the strongest associations with psychiatric morbidity.

**Table 3:** Independent associations between childhood maltreatment/family problems and psychiatric morbidity (*N* = 39,573).

	**Depression** ***N*** **= 411** **(1.0%)**	**Somatic disorder** ***N*** **= 2,429** **(6.1%)**	**Obsessive disorder** ***N*** **= 1,423** **(3.6%)**	**Suicide attempts** ***N*** **= 551** **(1.4%)**
	**OR (95%CI)**	**OR (95%CI)**	**OR (95%CI)**	**OR (95%CI)**
**Childhood maltreatment/disadvantages**				
Left-behind child *N* = 9,471 (23.9%)	1.55^***^ (1.27–1.91)	1.36^***^ (1.24–1.49)	1.30^***^ (1.16–1.46)	1.42^***^ (1.19–1.70)
Sexual abuse *N* = 1,147 (2.9%)	3.39^***^ (2.38–4.83)	1.78^***^ (1.47–2.16)	1.78^***^ (1.38–2.30)	3.22^***^ (2.35–4.43)
Physical abuse *N* = 10,339 (26.1%)	1.53^***^ (1.22–1.91)	1.24^***^ (1.12–1.37)	1.18^**^ (1.04–1.34)	1.96^***^ (1.61–2.38)
Emotional neglect *N* = 13,385 (33.8%)	2.82^***^ (2.24–3.55)	1.83^***^ (1.66–2.01)	2.05^***^ (1.81–2.32)	2.52^***^ (2.06–3.09)
**Family problems**				
Serious illnesses *N* = 4,963 (12.5%)	1.56^***^ (1.23–1.98)	2.28^***^ (2.06–2.52)	1.82^***^ (1.60–2.08)	1.73^***^ (1.41–2.13)
Relatives death *N* = 4,519 (11.4%)	1.02 (0.77–1.35)	2.06^***^ (1.85–2.30)	1.50^***^ (1.30–1.73)	1.34^*^ (1.06–1.68)
Family financial problems *N* = 11,553 (29.2%)	1.83^***^ (1.48–2.26)	2.60^***^ (2.38–2.83)	1.74^***^ (1.55–1.95)	1.63^***^ (1.36–1.94)
Conflicts between parents *N* = 10,023 (25.3%)	1.66^***^ (1.34–2.06)	2.81^***^ (2.58–3.07)	2.18^***^ (1.95–2.44)	2.42^***^ (2.02–2.88)

*Adjusted for sex, age, ethnic minority, low family income, childhood maltreatment/disadvantage, and family problems (all variables binary except age which is continuous)*.

* *p < 0.05,*

** *p < 0.01,*

*** *p < 0.001*.

Table 4 shows that the odds of association between experiencing stressful events indicating problems with adaptation and different categories of psychiatric morbidity were all substantially greater than those observed between childhood maltreatment/disadvantage and family problems shown in Table 3. Following adjustments for childhood maltreatment/disadvantage and family problems, all associations showed some attenuation, although this was generally small.

**Table 4:** Independent associations between stressful problems/events and psychiatric morbidity (*N* = 39,573).

	**Depression^**a**^** ***N*** **= 411** **(1.0%)**	**Somatic disorder^**a**^** ***N*** **= 2,429** **(6.1%)**	**Obsessive disorder^**a**^** ***N*** **= 1,423** **(3.6%)**	**Suicide attempts^**b**^** ***N*** **= 551** **(1.4%)**
	**OR (95%CI)**	**OR (95%CI)**	**OR (95%CI)**	**OR (95%CI)**
Study problem events	3.49^***^ (2.78–4.37)	4.15^***^ (3.78–4.56)	4.07^***^ (3.62–4.58)	3.33^***^ (2.78–3.99)
Study problem events^#^	2.73^***^ (2.13–3.50)	2.66^***^ (2.39–2.96)	3.08^***^ (2.70–3.52)	1.84^***^ (1.48–2.28)
Interpersonal problem events	3.53^***^ (2.80–4.44)	4.60^***^ (4.17–5.08)	3.86^***^ (3.41–4.37)	4.39^***^ (3.64–5.28)
Interpersonal problem events^#^	2.77^***^ (2.15–3.56)	2.85^***^ (2.54–3.19)	2.79^***^ (2.43–3.21)	2.51^***^ (2.01–3.14)
Adaptation problem events	4.54^***^ (3.62–5.68)	4.63^***^ (4.21–5.09)	3.10^***^ (2.73–3.51)	3.17^***^ (2.62–3.83)
Adaptation problem events^#^	3.72^***^ (2.92–4.73)	3.15^***^ (2.84–3.50)	2.24^***^ (1.96–2.57)	1.74^***^ (1.40–2.17)
All problem events	5.12^***^ (4.05–6.47)	5.37^^***^^ (4.88–5.92)	4.75^***^ (4.20–5.38)	4.08^***^ (3.40–4.91)
All problem events^#^	4.18^***^ (3.23–5.42)	3.44^***^ (3.08–3.85)	3.59^***^ (3.12–4.13)	2.18^***^ (1.74–2.74)

a *Adjusted for sex, age, ethnic minority, low family income, and other psychiatric morbidity except suicide attempt (binary variables except age which was continuous)*.

b *Adjusted for sex, age, ethnic minority, low family income (binary variables except age which was continuous)*.

# Additionally adjusted for childhood maltreatment/disadvantage and family problems (counts of event numbers). ^*^p < 0.05, ^**^p < 0.01,

*** *p < 0.001*.

### Interaction Analysis

We tested whether there were interactions between child maltreatment/disadvantage, current family problems, psychiatric morbidity, and outcome of high stress scores resulting from poor adaptation to university life. We combined each of the four categories of psychiatric morbidity, one or more of which had to be present for our binary psychiatric morbidity variable. We found significant interactions with poor adaptation score as outcome firstly, for interaction between child maltreatment and current family difficulties (Beta 0.56 95% CI [0.49–0.63]); secondly, for interaction between current family difficulties and psychiatric morbidity (Beta 0.57 95% CI [0.40–0.75]); and thirdly, for interaction between child maltreatment and psychiatric morbidity (Beta 0.33 95% CI [0.23–0.44]). We found one significant multiplicative interaction between current family problems and child maltreatment/disadvantage with a binary outcome of psychiatric morbidity (OR 1.06 [95% CI 1.03–1.10]).

### Explanatory Role of Psychiatric Morbidity in Associations Between Childhood Maltreatment, Family Stress, and Adaptation to University Life

Childhood maltreatment/disadvantage and family problems were both selected on the basis of their associations with adaptation to university scores (Table 2). Psychiatric morbidity was considered as a potential explanatory variable on the basis of significant associations with (a) child maltreatment/disadvantage, (b) ongoing family problems, and (c) adaptation to university scores.

**Figure 1:**
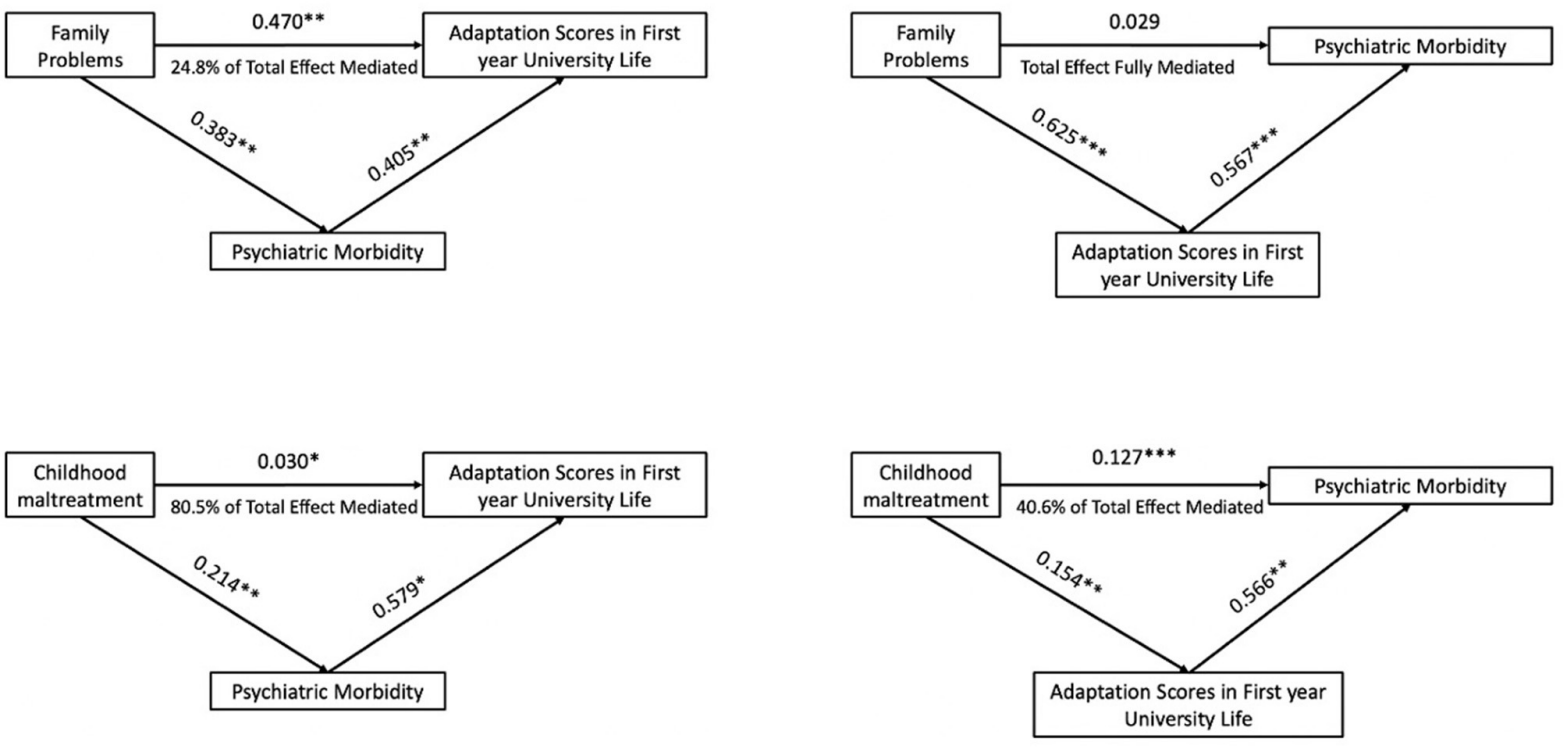
Explanatory models of associations between childhood maltreatment and family problems in adaptation stress of first year university students.

As shown in Figure 1, the pathway from experiencing childhood maltreatment/disadvantage to poor adaptation in the first year of university life showed significant indirect effects indicating that it was substantially, but not entirely, mediated by psychiatric morbidity. We therefore tested the pathway from childhood maltreatment/disadvantage to psychiatric morbidity which showed significant indirect effects, indicating that the pathway was partly mediated by poor adaptation to university life.

We next tested whether the pathway between current family problems and adaptation to university life was mediated by psychiatric morbidity. We found significant indirect effects, indicating part-mediation of the pathway by psychiatric morbidity. However, when we tested the pathway from family problems to psychiatric morbidity, the direct effects were no longer significant, indicating that the relationship between family problems and psychiatric morbidity was accounted for by the level of stress from adaptation problems during the first year of university life.

## Conclusion

The key findings of this study were that the impact on psychiatric morbidity of childhood maltreatment/disadvantage and recent family problems were both substantial but smaller than the overall impact of stress due to difficulties in adaptation during the first year of university life on psychiatric morbidity. At the same time, these risk factors were bidirectional and closely associated with each other so that psychiatric morbidity could also impact substantially on difficulty coping, leading to stress and poor adaptation among students. Those who had experienced child maltreatment and those currently experiencing difficulties in their families were both more likely to experience stress from coping with the pressures of university life and also psychiatric morbidity in their early adulthood. High levels of stress due to university life and pressure from their studies were greatest among younger and female students. We also found that childhood maltreatment increased the likelihood of students experiencing stress from ongoing family problems at the time they first entered university. This may have been because family problems in early life were more common and persistent among those who had experienced early maltreatment and/or had been left behind by their parents.

A further key finding was that no single category of psychiatric morbidity among the four conditions of depression, somatization, obsessive-compulsive disorder, or suicide attempts appeared to be more strongly associated with each of the three different domains of early maltreatment/disadvantage, family problems in early adulthood, and poor adaptation due to stress from university life. Although we carried out adjustments for other categories of psychiatric morbidity to measure independent effects and found the odds of association were greater for certain associations with depression, these differences were small and not statistically significant.

When we compared odds of independent associations between adverse childhood experiences, current family problems, and stress due to poor adaptation to undergraduate life on psychiatric morbidity, we found that stress from poor adaptation was most strongly associated. Finally, after adjusting for potential confounding effects of both child maltreatment/disadvantage and family problems, associations between stress due to poor adaptation and psychiatric morbidity showed only small attenuation, and with no attenuation in the case of depression. Our interaction analyses showed multiplicative effects of childhood risk factors and ongoing family problems on both poor adaptation to university life due to stress and also on psychiatric morbidity. Similarly, we found multiplicative effects of both psychiatric morbidity and childhood maltreatment, and psychiatric morbidity and family problems, on stress from poor adaptation as outcome. However, we did not find interactions between stress due to poor adaptation and these two areas of risk factors with psychiatric morbidity as outcome.

Our original aim had been to test two hypothesized pathways, one from childhood experiences of maltreatment and parental separation through being left behind, the other from recent distressing family problems, both leading to increased prevalence of psychiatric morbidity and resulting in poor adaptation. The first pathway from childhood maltreatment/disadvantage was largely confirmed in a proportion of students. Negative childhood experiences were associated with greater risk of psychiatric morbidity in early adulthood which substantially mediated stress arising from poor adaptation to university. We also observed that childhood maltreatment/disadvantage together with psychiatric morbidity had multiplicative effects on poor adaptation. However, we found that associations between current family problems showed little evidence of mediation by psychiatric morbidity in their association with stress due to poor adaptation. In contrast, the associations we initially observed between family problems and psychiatric morbidity were entirely mediated by stress due to poor adaptation.

### Stress and Adaptation to College Life

Childhood adversity is associated with the development, maintenance, and recurrence of a range of psychiatric disorders later in life (35), corresponding to our findings for a subgroup of students. However, apart from the experience of moving from home, which may have affected students in the sample who had been left behind in rural areas by their parents during childhood, the predisposing effects of early maltreatment are likely to have had a general rather than specific effect. Nevertheless, some life experiences, such as early trauma, are thought to increase vulnerability to further stressful and traumatic events occurring in adulthood (36) as well as greater sensitivity to these events when they occur (37, 38), thereby increasing risk through different mechanisms which increase vulnerability to psychiatric disorders. In the case of family problems at the time of university entry, it is probable that the latter represented unresolved difficulties between close relatives that had been present during their childhood leading to maltreatment and disadvantage and had persisted into our participants' early adulthood.

Stressful life events involving students' families were associated with psychiatric morbidity, but these effects were entirely mediated by those of stress due to poor adaptation to university life. The most prevalent items reflected stress from academic study. Support from friends at college and positive feelings of institutional attachment have been identified as protective factors in the case of bereavement and positively associated with academic and social adjustment (39). These may have operated to reduce the impact of family problems. However, our findings suggested that any protective effects were unlikely to be effective when students were experiencing severe difficulties with adaptation to college.

Studies of medical students have suggested that possible causes of depression and suicidal ideation are likely to include stress and anxiety secondary to the competitiveness of medical school (40). Our findings confirm the importance of competitiveness and inter-personal relationships, but also suggest that the probable causes of their psychiatric morbidity included stress from the very substantial burden of work that is typically placed on freshmen at Chinese universities.

## Strengths and Limitations

Strengths of this study include the large sample size and comprehensive range of stressful life events to assess adaptation status at university. However, the study had limitations. First, cross-sectional data cannot establish causal relationships. The relationship between childhood maltreatment/stressful family factors, stressful life events, and psychiatric morbidity should therefore be investigated in more comprehensive longitudinal studies. Second, our findings may not generalize to a general population of young persons. We do not know whether they experienced fewer childhood traumas and more protective factors associated with more family financial support, or whether they experienced more stress during their upbringing associated with their parents accumulating wealth by migration within China. Third, psychiatric morbidity was assessed entirely by self-report instruments. Although most studies of psychiatric morbidity in students use self-report, more use of standardized interviews is recommended (3). Fourthly, measuring childhood maltreatment using simple yes/no responses on physical abuse, sexual abuse, neglect, and being left behind was somewhat simplistic. Combining responses at each level of severity on the questionnaire did not permit examination of the severity of these experiences on outcomes in our analyses. This may explain why we did not find more associations between childhood maltreatment and current measures of adaptation to university. In addition, asking these questions retrospectively may have resulted in recall bias. It also assumes that the students had full understanding of the variables we wished to measure, and this may have been incorrect. Nevertheless, previous research indicates that retrospective assessment of childhood adversity has good reliability, although these studies tend to include more questions and additional information (41). It will be important to measure maltreatment history more precisely in future investigations.

## Implications

The study provides a potential model of clinical consultation for student health services when dealing with a student presenting with distress in the context of their studies. We have chosen high levels of stress due to study problems, interpersonal difficulties, and difficulty adapting to a new life in the university environment as our primary outcomes in this investigation. However, levels of stress can be affected by the relationship with and ongoing difficulties between the student and their families. The student may have unresolved issues from their childhood and may even be traumatized by their early experiences. In some cases, the student may have psychopathology of sufficient severity to constitute a diagnosis of psychiatric morbidity requiring treatment intervention. Taken together, this means that a clinical evaluation should cover each of the four main areas, including stressful life events at the university, current family problems, childhood maltreatment and disadvantage, and whether symptoms of psychopathology are present to gain a full understanding of the presenting problem. Each of these components may be the main presenting problem in a consultation but are potentially interlinked and can be bidirectional. These considerations are important in determining the most appropriate interventions.

Our findings are new and have implications for delivering prevention and interventions to students who experience severe stress at university. Previous surveys have shown that only a minority of distressed university students seek professional help, possibly due in part to lack of time and also to stigma related to mental health (42). Our findings must be considered in this background and highlight the need for more students to access services. They also highlight the need for student healthcare services to screen for pre-existing traumatic experiences in childhood and ongoing family problems (problems students bring with them), including maltreatment and the complex disadvantages conveyed on some individuals by being left behind children. For example, a study has suggested that the length of time the child has been separated from parents is important in increasing risk of psychiatric morbidity in adulthood, with some additional effect from critical timing of separation according to age (12). However, left behind children are also more likely to experience moves of residence and residential moves convey increased and independent risks of future psychiatric morbidity (43). These early experiences should be considered risk factors for future stress in university adaptation, but also for increased risk of distress due to ongoing and unresolved family problems at the time of entry to university. Some students may have experienced sufficiently severe impact from childhood traumas to require primary psychotherapeutic intervention for psychiatric morbidity deriving these early experiences, particularly sexual abuse. This may be revealed by the student for the first time to healthcare services when coming to university. Our findings also indicate the need to screen for ongoing problems of poor parental relationships, financial difficulties, and illness in families of origin which may impact the student's ability to adapt to the new environment but which the student may feel powerless to do anything about and where they are likely to remain dependent on their family for emotional and financial support. Nevertheless, our findings also suggest the most important area of assessment may be the level of stress due to current adaptation problems with their studies, relationships with other students and teachers, and coping in a new environment rather than primarily focusing on problems that students bring with them to university. Overall, when we compared effects of different domains, it was problems encountered at university that appeared to have the strongest impact on psychiatric morbidity. However, it is also important to consider the bi-directionality of these many interlinked factors, as indicated by our study. For example, a meta-analysis of studies that examined the relationship between mental health status and academic performance revealed that the overall intensity of general stress has a negative impact on academic performance and social functioning (44).

Although it is clearly necessary to screen students for early trauma, stress management, which is an easier intervention, may be a generally more effective approach to promoting college students' mental health than trauma-focused interventions. Psycho-education, relaxation, and cognitive monitoring/restructuring are the most common elements among these interventions (45). Meta-analysis has indicated that for common mental disorders, CBT and mindfulness-based interventions are effective for both depression and generalized anxiety disorder (GAD), and attention/perception modification is effective for GAD. Other interventions such as art, exercise, and peer support appear effective for both depression and GAD (46), including exercise for those at risk for depression (47). For those who are not thought to require medication in the case of less severe psychiatric morbidity, there is some evidence that computer-delivered and internet-based psychological interventions, which are acceptable to young adults and can meet high-level demands, in particular cognitive behavioral therapy (CBT), may alleviate depression and anxiety in university students (48, 49). Linking surveys such as ours with these interventions may be a helpful future approach.

Finally, our results suggest it is important to consider prevention and that there should be special focus on younger and female students. Screening at college entrance has been proposed to identify students at risk of depression onset, with the strongest predictors identified as history of childhood-adolescent trauma, stressful experiences in the past 12 months, and other mental disorder, particularly prior suicidal behaviors (50). It has also been proposed that schools and universities should enhance social and emotional learning to promote resilience in at-risk students (51). In addition, if the primary problem appears to be difficulty in adaptation to university life, problem-focused styles of coping for psychological adjustment may be beneficial in modifying a reactive style which has been found to have a direct link with psychological symptoms (52). However, our findings suggest that the university physical and social environment should also be considered and evaluated, together with the burden on students of their studies. This includes whether there is a need to recommend restructure of curricula and student evaluations.

## Data Availability Statement

The raw data supporting the conclusions of this article will be made available by the authors, by reasonable request.

## Ethics Statement

The studies involving human participants were reviewed and approved by Medical Ethics Committee of West China Hospital of Sichuan University. Written informed consent from the participants' legal guardian/next of kin was not required to participate in this study in accordance with the national legislation and the institutional requirements.

## Author Contributions

YaZ, WT, QW, WD, XM, YM, ML, WG, and TL designed the study. XLi, HS, LZ, HW, TC, and QL were responsible for data collection and cleaning. YiZ and XLiu performed the analyses. JC, YiZ, and TL drafted the manuscript. All authors commented and approved for submission.

## Funding

This work was partly funded by National Natural Science Foundation of China (TL, 81630030 and TL, 81920108018), Special Foundation for Brain Research from Science and Technology Department of Guangdong (2018B030334001), and 1.3.5 Project for disciplines of excellence, West China Hospital of Sichuan University (TL, ZY2016103 and TL, ZY2016203).

## Conflict of Interest

The authors declare that the research was conducted in the absence of any commercial or financial relationships that could be construed as a potential conflict of interest.

## Publisher's Note

All claims expressed in this article are solely those of the authors and do not necessarily represent those of their affiliated organizations, or those of the publisher, the editors and the reviewers. Any product that may be evaluated in this article, or claim that may be made by its manufacturer, is not guaranteed or endorsed by the publisher.
